# Knockdown of CENPF inhibits the progression of lung adenocarcinoma mediated by ERβ2/5 pathway

**DOI:** 10.18632/aging.202303

**Published:** 2021-01-10

**Authors:** Tang Hexiao, Bai Yuquan, Xiong Lecai, Wei Yanhong, Shen Li, Hu Weidong, Xu Ming, Zhou Xuefeng, Pan Gaofeng, Zhang Li, Zhu Minglin, Tang Zheng, Yang Zetian, Zhou Xiao, Cai Yi, Michael Lanuti, Zhao Jinping

**Affiliations:** 1 Department of Thoracic Surgery, Zhongnan Hospital of Wuhan University, Wuhan, China; 2 Department of Geriatrics, Zhongnan Hospital of Wuhan University, Wuhan, China; 3 Division of Thoracic Surgery, Massachusetts General Hospital, Harvard Medical School, Boston, MA 02114, USA

**Keywords:** centromere protein F (CENPF), estrogen receptor beta, lung adenocarcinoma (LUAD), WGCNA package, non-small cell lung cancer (NSCLC)

## Abstract

Many studies have reported that estrogen (E2) promotes lung cancer by binding to nuclear estrogen receptors (ER), and altering ER related nuclear protein expressions. With the GEO database analysis, Human centromere protein F (CENPF) is highly expressed in lung adenocarcinoma (LUAD), and the co-expression of CENPF and ERβ was found in the nucleus of LUAD cells through immunofluorescence. We identified the nuclear protein CENPF and explored its relationship with the ER pathway. CENPF and ERβ2/5 were related with T stage and poor prognosis (P<0.05). CENPF knockout significantly inhibited LUAD cell growth, the tumor growth of mice and the expression of ERβ2/5 (P<0.05). The protein expression of CENPF and ERβ2/5 in the CENPF-Knockdown+Fulvestrant group was lower than CENPF- Negative Control +Fulvestrant group (P=0.002, 0.004, 0.001) in A549 cells. The tumor size and weight of the CENPF-Knockdown+Fulvestrant group were significantly lower than CENPF- Negative Control +Fulvestrant group (P=0.001, 0.039) in nude mice. All the results indicated that both CENPF and ERβ2/5 play important roles in the progression of LUAD, and knockdown CENPF can inhibit the progression of LUAD by inhibiting the expression of ER2/5. Thus, the development of inhibitors against ERβ2/5 and CENPF remained more effective in improving the therapeutic effect of LUAD.

## INTRODUCTION

Lung cancer is one of the most commonly diagnosed cancers and a leading cause of cancer related mortality [1]. The global incidence and mortality of lung cancer is increasing significantly [2]. In addition to environmental exposures such as smoking, the growth factor pathway and hormonal regulation also play critical roles in the carcinogenesis of lung cancer [3].

Estrogen receptors (ERs) belong to the nuclear receptor steroid superfamily, and are closely linked to hormonal regulations. ERs are classified into two subtypes, ERα and ERβ, and these have different tissue distributions and biological effects in various tumor types [4]. Previous studies have revealed that estrogen activates the transcription of target genes by binding directly to ERα and ERβ [5]. Numerous studies have found that ER interacts with other transcription factors such as activator protein 1 (AP-1), specificity protein 1 (SP-1), interleukin 6 (IL-6) and epidermal growth factor (EGF) through protein-protein interactions [6]. Analysis of four lung cancer gene chips revealed that the nuclear protein gene, human centromere protein F (CENPF), is highly expressed in lung adenocarcinoma (LUAD). Furthermore, the expression of CENPF and ERβ2/5 in LUAD patients have been shown to be correlated to TNM staging, providing a basis for exploring the interactions between CENPF and ERβ2/5. Rattner et al. demonstrated that CENPF is involved in mitosis and tumor proliferation [7]. The full-length molecular weight of CENPF is 367 KDa and contains 3,210 amino acids [8]. In prostate cancer, CENPF has been shown to predict survival and tumor metastasis [9]. CENPF is directly associated with disease outcomes after undergoing gene amplification [10]. However, the role of CENPF in the progression of LUAD still remained unclear.

A large number of studies have shown that estrogen (E2) promotes the progression of lung cancer by binding to nuclear ERs [11]. Our previous studies have shown that among the five types of ERs, lung cancer tissues express found the ERβ1/2/5 [12, 13]. As a full-length fragment of ERβ subtype, ERβ1 is responsible for the action of other subtypes [14], and so the role of ERβ2/5 in the progression of LUAD remained the main focus of our current research.

Based on estrogen gene signaling pathway, a high co-expression of CENPF and ERβ2/5 showed association with the clinicopathological features and prognosis of LUAD patients. CENPF is hypothesized as one of the key nuclear proteins in estrogen gene signaling pathway. Therefore, this study mainly explored the relationship between CENPF and ERβ2/5, and also explored their role alone in the development of LUAD. This study will provide a better understanding of ERs gene signaling pathway and improve the prognosis of LUAD patients.

## RESULTS

### Bioinformatics analysis of lung cancer datasets and the determination of CENPF

**Figure 1:**
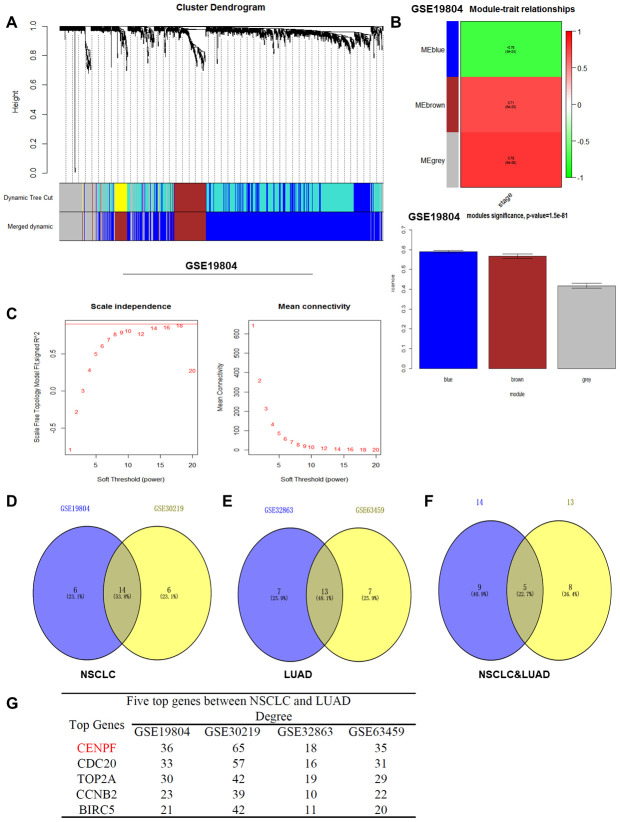
**WGCNA analysis and determination of the CENPF gene.** (**A**) Dendrogram of differentially expressed genes clustered based on a dissimilarity measure (1-TOM). (**B**) Heat map distribution histogram of differential genes for modules related to NSCLC staging in GSE19804 (The same results in the GSE30219, GSE32863, GSE63459 databases are shown in Supplementary Figure 1). (**C**) Analysis of the scale-free fit index for various soft-thresholding power (β) and analysis of the mean connectivity for various soft-thresholding power (GSE19804). (**D**) There are 14 gene differential expressions in the NSCLC staging modules. (**E**) There are 13 gene differential expressions in the LUAD staging modules. (**F**) In the four datasets, there were 5 overlapping genes that were significantly differentially expressed between NSCLC and LUAD. (**G**) The degree values of the five key genes in different datasets.

Differential genes with similar expression pattern in each dataset (GSE19804, GSE30219, GSE32863, GSE63459) were used (Figure 1A–1C, Supplementary Figure 1A–1I, β=5, 6, 5, 7) [15]. The module genes obtained by the four datasets showed significant association with TNM staging of lung cancer, which included the brown module of GSE19804 (n=185), the turquoise module of GSE30219 (n=413), the yellow module of GSE32863 (n=63) and the yellow module of GSE63459 (n=160) (Figure 1B; Supplementary Figure 1B, 1E, 1H; Supplementary Table 1). By overlapping the module genes, five key genes including CENPF, CDC20, TOP2A, CCNB2 and BIRC5 were obtained (Figure 1D–1G). Finally, based on the central degree of the five key genes in different datasets and relevant literature [9, 16], the hub gene was identified as CENPF.

### CENPF is highly expressed in LUAD and negatively correlated with the prognosis of LUAD patients

**Figure 2:**
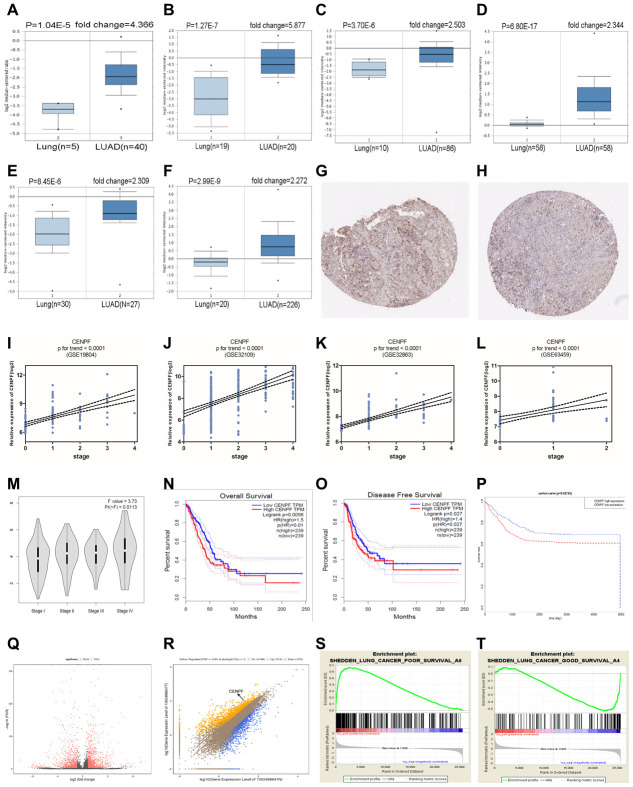
**CENPF is upregulated in LUAD and is related with TNM staging and prognosis of LUAD patients.** (**A**–**F**) Oncomine database results show that CENPF expression is significantly up-regulated in LUAD. (The corresponding P value and fold change are given above the picture). (**G**, **H**) The Human Protein Atlas database indicates that CENPF is strongly expressed in LUAD. G: LUAD (patient ID.4923, male, 57); H: normal lung tissue (patient ID. 4208, male, 75). (**I**–**L**) Analyzes the relationship between CENPF and LUAD staging based on four datasets. (**M**) Verify the correlation between the expression of CENPF and the pathological stage of LUAD (based on TCGA data in GEPIA). N-P: Survival analysis. (**N**, **O**) Kaplan Meier curves of OS (overall survival), DFS (Disease-free survival) in a cohort of LUAD stratified by CENPF expression. (**P**) Survival curves of CENPF gene in LUAD patients based on TCGA database. (**Q**) RNA sequencing analysis of volcano maps. (**R**) RNA sequencing results indicate that CENPF is highly expressed in LUAD tissues. Orange represents a high expression of the gene in LUAD, and blue represents a low expression of the gene in LUAD. (**S**, **T**) The CENPF gene was analyzed using Gene Set Enrichment Analysis (GSEA). The positive expression of the CENPF is related with a low prognosis in LUAD patients.

CENPF was highly expressed in LUAD when compared to normal lung tissues (Figure 2A–2H). The expression of CENPF was positively correlated to the TNM staging of LUAD (P<0.01, Figure 2I–2M). Additionally, high expression of CENPF was negatively correlated with overall survival and disease-free survival in LUAD patients (P=0.01, 0.027, 0.0267, Figure 2N–2P). The result of RNA-Seq included 1515 high expressed genes and 1370 low expressed genes in LUAD patients (Figure 2Q). CENPF was included in high expressed genes (Figure 2R, P<0.05) and was associated with poor prognosis in LUAD patients (Figure 2S, 2T).

### Expression of CENPF, ERβ, ERβ1, ERβ2 and ERβ5, and its relationship with TNM staging and prognosis of LUAD patients

**Figure 3:**
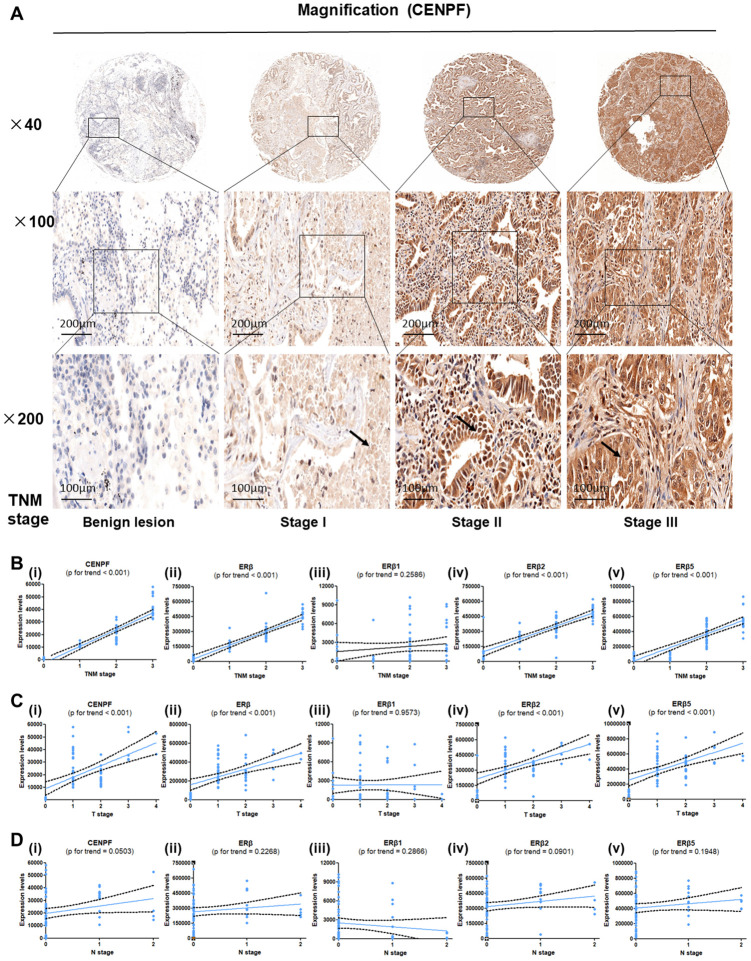
**Expression of CENPF, ERβ, ERβ2 and ERβ5 are associated with T stage and TNM stage in LUAD patients.** (**A**) Tissue microarray (TMA) was used to analyze the expression of CENPF in benign lung lesions and different TNM staging tissues of LUAD. The magnification of each slice is 40×, 100×, 200× in order. (**B**–**D**) Analysis of the relationship between the expression of CENPF, ERβ, ERβ1, ERβ2 and ERβ5 and the TNM staging or T stage or N stage of LUAD. The corresponding P value is marked above the picture.

The expressions of CENPF, ERβ, ERβ1, ERβ2 and ERβ5 in different TNM stages of LUAD and benign primary lesions (BPL) were examined (Figure 3A, Supplementary Figure 2A–2D). The results revealed that CENPF, ERβ, ERβ2 and ERβ5 were highly expressed in LUAD and showed a positive correlation with TNM staging and T grade of LUAD patients (P<0.001, Figure 3B, 3C), but showed no association with nodal involvements (Figure 3D). Moreover, analysis of high expression of CENPF in LUAD patients showed significant association of CENPF with shorter survival rate (Table 1).

**Table 1:** CENPF expression in the lung adenocarcinoma. Bold numbers represent statistical significance.

**Variables**	**case (n=56)**	**high**	**low**	**P value**
Gender				
Male	37	14	23	0.389
Female	19	5	14	
Age(year)				
>65	21	7	14	0.942
≤65	35	12	23	
Tumor size(cm)				
≥5	12	8	4	**0.007**
<5	44	11	33	
Smoking				
Smoking	22	8	14	0.757
No-smoking	34	11	23	
TNM stage				
I	9	0	9	**0.019**
II-III	47	19	28	
Tgrade				
T1-T3	54	17	37	**0.044**
T4	2	2	0	
N grade				
N0	40	15	25	0.372
N1-N2	16	4	12	
Relapse				
Relapse	5	4	1	**0.041**
No-relapse	51	15	36	
Survival months	27.39±7.54	33.41±13.86	**0.048**

### CENPF knockdown inhibits biological effects of LUAD cells

**Figure 4:**
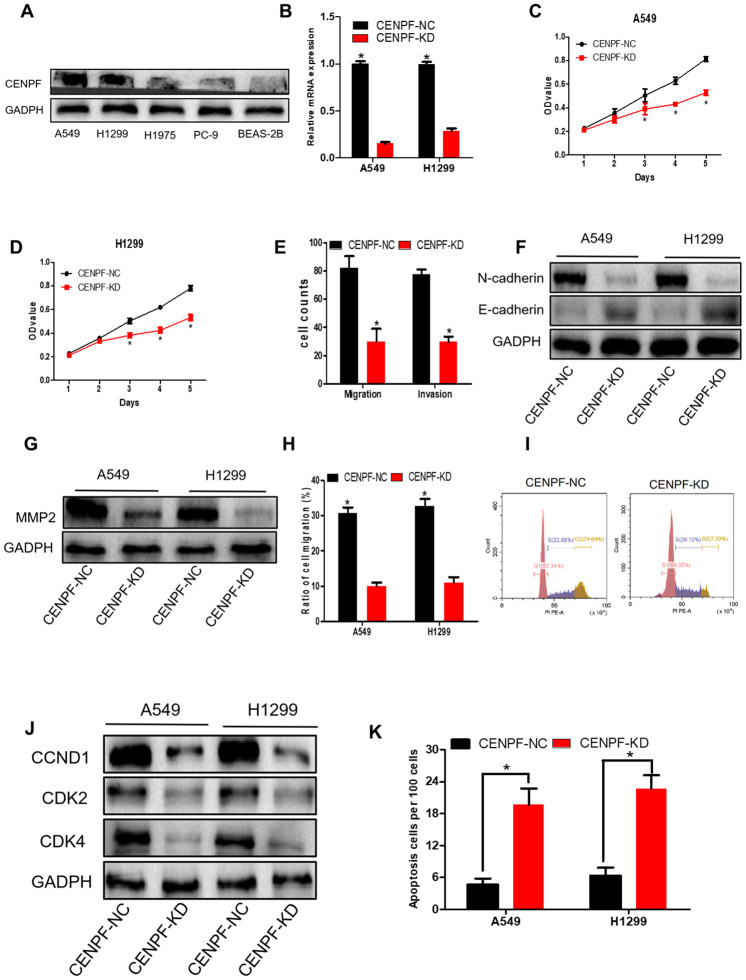
**Knockdown of CENPF inhibits cell proliferation, migration, invasion and increases apoptosis of LUAD cells.** (**A**) The protein level of CENPF in A549 and H1299 cell lines were higher than in normal cell lines BEAS-2B and other LUAD cells. GAPDH served as the internal control. (The corresponding gray value are shown in Supplementary Figure 3). (**B**) The knockdown efficiency of LV-CENPF sh or LV-NC transfected with A549 and H1299 cells was verified by RT-qPCR. *P < 0.05 vs CENPF-KD. (**C**, **D**) MTT showed that CENPF knockdown suppressed the proliferative viability of cells in A549 and H1299 cells. *P < 0.05 vs CENPF-KD. (**E**) Migration assays and invasion assays revealed that CENPF-KD decreased cell migration and invasion abilities of A549. (**F**, **G**) The related protein E-cadherin was significantly increased (P=0.009, Figure 4F; The corresponding gray value are shown in Supplementary Figure 3G), and N-cadherin and MMP2 were significantly decreased when compared with NC group (P=0.004, 0.012; The corresponding gray value are shown in Supplementary Figure 3H). (**H**) Quantified histograms of scratch experiment of A549 and H1299. (**I**) The cell percentage and DNA content were significantly increased in the G1 phase in the CENPF-KD group(P=0.011). (**J**) The expression of CCND1, CDK2 and CDK4 was significantly lowered in CENPF-KD group (P=0.022, 0.001, 0.002; The corresponding gray value are shown in Supplementary Figure 3M). (**K**) CENPF knockdown increased apoptosis of A549 and H1299 cell lines (P=0.001, 0.001). Each experiment was performed in triplicate and repeated three times. P values were calculated with two-tailed unpaired Student’s t test.

Compared with BEAS-2B and other LUAD cells, CENPF was highly expressed in A549 and H1299 cells (P<0.05, Figure 4A; The corresponding gray value are shown in Supplementary Figure 3A, 3B). RT-PCR and cellular immunofluorescence confirmed that CENPF knockdown (KD) was seen in 70% in A549 and H1299 cells (Figure 4B; Supplementary Figure 3C). Cell proliferation of CENPF-KD group was significantly weaker than the control (NC) group from day 3 in stable CENPF-deficient cell lines A549 and H1299 (P=0.007, 0.000, Figure 4C, 4D). At the same time, cells in CENPF-KD group demonstrated less Ki67 staining than NC group (Supplementary Figure 3D). In addition, the invasion and migration of A549 cells in the CENPF-KD group were significantly decreased (P=0.000, 0.000, Figure 4E; Supplementary Figure 3E, 3F) while E-cadherin expression was significantly increased (P=0.009, Figure 4F; The corresponding gray value are shown in Supplementary Figure 3G), and N-cadherin and MMP2 were significantly decreased when compared with NC group (P=0.004, 0.012, Figure 4F, 4G; Supplementary Figure 3G, 3H). A similar trend was obtained in the stable CENPF-deficient cell line H1299 (P<0.01, Supplementary Figure 3E–3H). Scratch experiment also demonstrated similar results (P=0.000, 0.000, Figure 4H; Supplementary Figure 3I). In A549 cells, the cell percentage and DNA content were significantly increased in the G1 phase in the CENPF-KD group when compared with NC group (P=0.011, Figure 4I; Supplementary Figure 3J). At the same time, the expression of CCND1, CDK2 and CDK4 was significantly lowered in CENPF-KD group (P=0.022, 0.001, 0.002, Figure 4J; The corresponding gray value are shown in Supplementary Figure 3M). Similar results were obtained in the stable CENPF-deficient cell line H1299 (P<0.05, Supplementary Figure 3K–3M). The CENPF-KD group also showed a significant increase in apoptosis when compared with NC group (P=0.001, 0.001, Figure 4K; Supplementary Figure 3N).

### CENPF Knockdown inhibits biological effects of LUAD cells mediated by ERβ2/5 pathway

**Figure 5:**
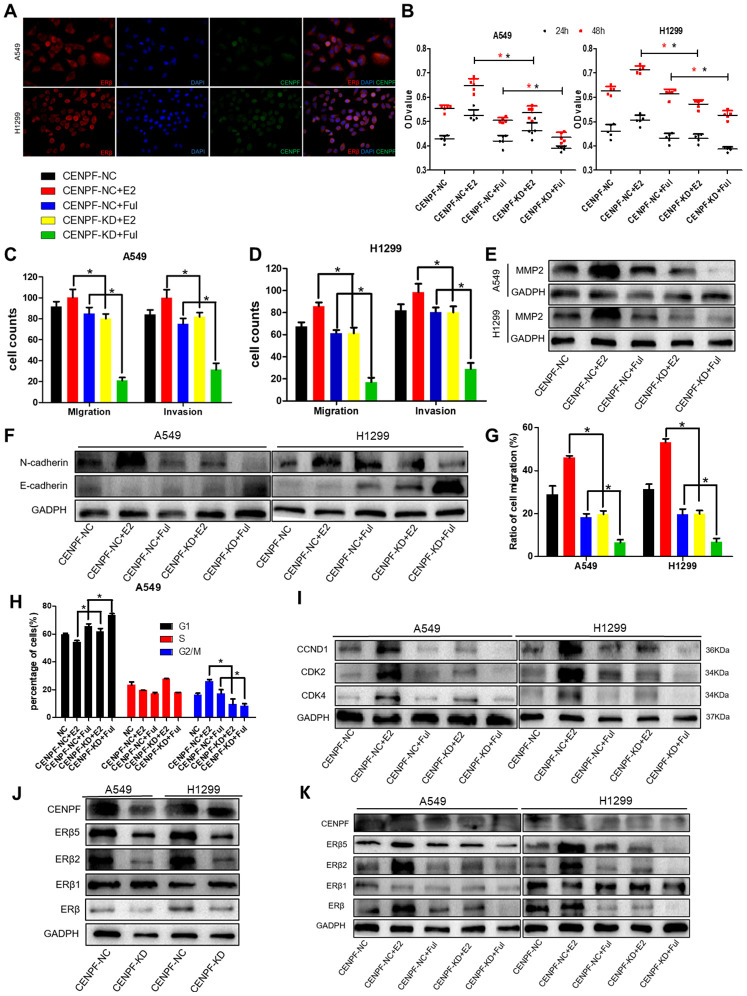
**Knockdown of CENPF inhibits proliferation, invasion and migration of LUAD cells via the ERβ2/5 pathway.** (**A**) Immunofluorescence showed the co-localization of CENPF and ERβ in A549 and H1299 cells (400 x). (**B**) Cell proliferation assays of different grouped cells at specific times in A549 and H1299 cells. (**C**, **D**) Corresponding quantified histograms of migration and invasion in A549 and H1299 cells. The invasion and migration of cells in CENPF-KD+E2 group were significantly reduced when compared with CENPF-NC+E2 group. (**E**, **F**) Protein expression of MMP2, N-cadherin and E-cadherin in A549 and H1299 cells (The corresponding gray value are shown in Supplementary Figure 4B–4D): The expression of MMP2 and N-cadherin were significantly decreased in CENPF-KD+E2 group when compared with CENPF-NC+E2 group. (**G**) Scratch experiment showed that the migration of CENPF-KD+E2 group was significantly lower than CENPF-NC+E2 group in A549 and H1299 cells (P=0.000, 0.000). (**H**) Corresponding quantified histograms of the A549 cells at different stages of the cell cycle (G1, S and G2/M). (**I**) Protein expression of CCND1, CDK2 and CDK4 in A549 and H1299 cells (The corresponding gray value are shown in Supplementary Figure 4F). *P < 0.05. (**J**) Knockdown of CENPF inhibited the expression of ERβ2/5 *in vitro*. (The corresponding gray value are shown in Supplementary Figure 5A). (**K**) Protein expression of CENPF, ERβ, ERβ1, ERβ2 and ERβ5 *in vitro* experiment after treated with E2 and Ful treatment (The corresponding gray value are shown in Supplementary Figure 5C). *P < 0.05. P values were calculated with two-tailed unpaired Student’s t-test, or one-way analysis of variance.

CENPF and ERβ were co-localized in the nucleus of LUAD cells (Figure 5A). To investigate the biological effects of CENPF knockdown in LUAD cells mediated by ERβ signaling pathway, the cells were divided into CENPF-NC, CENPF-NC+E2, CENPF-NC+Ful, CENPF-KD+E2, and CENPF-KD+Ful. Cell proliferation in CENPF-KD+Ful group was significantly lower than CENPF-NC+Ful group at 48 hours (P=0.000, 0.000, Figure 5B). In A549 cells, the invasion and migration in CENPF-KD+E2 group were significantly reduced when compared with CENPF-NC+E2 group (P=0.000, 0.000, Figure 5C; Supplementary Figure 4A). Similarly, the expression of MMP2 and N-cadherin were significantly decreased in CENPF-KD+E2 group when compared with CENPF-NC+E2 group (P=0.002, 0.016, Figure 5E, 5F; The corresponding gray value are shown in Supplementary Figure 4B–4D). Similar results were obtained in stable CENPF-deficient cell line H1299 (P<0.01, Figure 5D–5F, Supplementary Figure 4A–4D). Scratch experiment also showed that the migration of CENPF-KD+E2 group was significantly lower than the CENPF-NC+E2 group (P=0.000, 0.000, Figure 5G; Supplementary Figure 4E). Flow cytometry analysis showed that the percentage of cells in G2/M phase in CENPF-KD+E2 group was significantly reduced than CENPF-NC+E2 group in A549 and H1299 cells (P=0.001, 0.021, Figure 5H; Supplementary Figure 4G–4I). At the same time, the protein expression of CCND1, CDK2 and CDK4 in CENPF-KD+E2 group demonstrated a significant decrease (P=0.003, 0.008, 0.006, P=0.043, 0.004, 0.005, Figure 5I. The corresponding gray value are shown in Supplementary Figure 4F).

The effect of CENPF knockdown on the expression of ERβ2/5 was examined *in vitro* in CENPF-NC and CENPF-KD groups. The protein expression of ERβ2/5 in CENPF-KD group was significantly lower than the CENPF-NC group (P_A549_=0.013, 0.000; P_H1299_=0.006, 0.002, Figure 5J; The corresponding gray value are shown in Supplementary Figure 5A). We further explored the effect of CENPF knockdown on the expression ERβ2/5 under the action of E2 and Ful *in vitro*. The results revealed that the protein expression of CENPF and ERβ2/5 in CENPF-KD+Ful group was significantly lower than CENPF-NC+Ful group (P=0.002, 0.004, 0.001, Figure 5K; The corresponding gray value are shown in Supplementary Figure 5C) in A549 cells. Similar results were obtained in stable CENPF-deficient cell line H1299 (P < 0.01, Figure 5J, 5K; Supplementary Figure 5C).

### Knockdown of CENPF can inhibit ERβ2/5 pathway-mediated tumor growth *in vivo*

**Figure 6:**
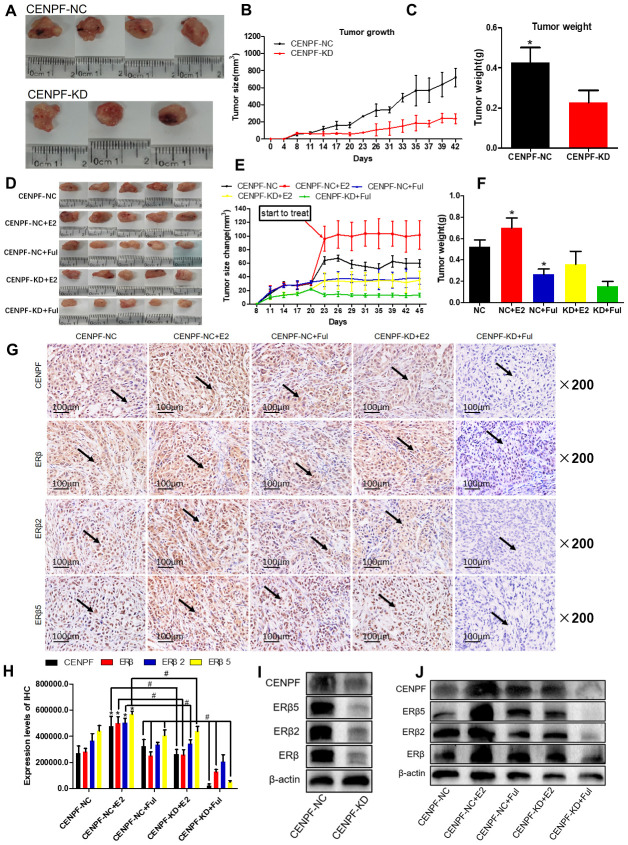
**Knockdown of CENPF can inhibit ERβ2/5 pathway-mediated tumor tissue growth *in vivo*.** (**A**) Pictures of mice tumor tissues after resection. (**B**, **C**) Analysis of tumor size and tumor weight. *P < 0.05. (**D**) Tumor images of nude mice. (**E**, **F**) Statistical analysis of tumor size and tumor weight. (**G**, **H**) Immunohistochemical analysis of the expression of CENPF, ERβ, ERβ2 and ERβ5 in nude mice tumor tissues and corresponding quantified histograms. (**I**) Knockdown of CENPF inhibited the expression of ERβ2/5. (The corresponding gray value are shown in Supplementary Figure 5B). (**J**) Protein expression of CENPF, ERβ, ERβ1, ERβ2 and ERβ5 *in vivo* experiment after treated with E2 and Ful treatment (The corresponding gray value are shown in Supplementary Figure 5F). *P < 0.05. P values were calculated with two-tailed unpaired Student’s t-test, or one-way analysis of variance.

The tumor weight and size in the CENPF-KD group were lower than NC group (P<0.001, 0.001, Figure 6A–6C). The results of immunohistochemistry also showed that the expression of CENPF and ERβ2/5 was significantly decreased in CENPF-KD group when compared to the NC group (P=0.000, 0.000, 0.000, Supplementary Figure 5D, 5E).

In the lung cancer model of nude mice, tumor size and weight in the CENPF-KD+Ful group were significantly lowered than CENPF-NC+Ful group (P=0.001, 0.039, Figure 6D–6F; Supplementary Figure 5G). Immunohistochemistry staining demonstrated that the expression of CENPF and ERβ2/5 in CENPF-KD+E2 group was significantly lower than that in CENPF-NC+E2 group (P=0.000, 0.000, 0.000, Figure 6G, 6H).

Similar to the *in vitro* experiments, the effect of CENPF knockdown on the expression of ERβ2/5 was examined in CENPF-NC and CENPF-KD groups, and examined under the action of E2 and Ful *in vivo*. The protein expression of CENPF and ERβ2/5 in CENPF-KD group was lower than CENPF-NC group *in vivo* (P=0.024, 0.020, 0.003, Figure 6I; The corresponding gray value are shown in Supplementary Figure 5B). The protein expression of CENPF and ERβ2/5 was significantly decreased in CENPF-KD+Ful group when compared with CENPF-NC+Ful group in mice tumor tissues (P=0.020, 0.004, 0.002, Figure 6J; The corresponding gray value are shown in Supplementary Figure 5F).

## DISCUSSION

Lung cancer is one of the most common malignancies and poses as a major health crisis globally. Targeted therapy for cell proliferation-related pathways and hormone therapy for lung cancer are considered important treatment modalities [17]. ER signaling pathways mainly included gene signaling and non-gene signaling pathways [18]. Among them, the gene signaling pathway involves the integration of estrogen with ER and the association with estrogen response element (ERE) to promote the recruitment of RNA polymerase II and regulate gene transcription. During this process, different combinations of synergistic activators determine the specificity of ER for activating the target genes [5]. Therefore, the key genes involved in TNM staging of LUAD in the four lung cancer datasets (GSE19804, GSE30219, GSE32863, GSE63459) were analyzed by the "WGCNA" R package (Figure 1A–1C; Supplementary Figure 1, Supplementary Table 1). Ultimately, CENPF was found by overlapping the key genes (Figure 1D–1G).

High expression of CENPF was shown to correlate with the malignant progression and poor prognosis in patients with LUAD. Studies have shown that CENPF has been up-regulated in a variety of malignancies, including nasopharyngeal carcinoma, esophageal squamous cell carcinoma and prostate cancer [19–21]. The expression of CENPF in LUAD was detected by the Oncomine database. Similarly, RNA-Seq data showed high expression of CENPF in LUAD (Figure 2A–2F, 2Q, 2R). This result was further confirmed by analyzing the expression of CENPF according to LUAD staging in the four datasets and in *in vitro* experiment (Figure 2I–2L; Figure 6G, 6H), indicating that high expression of CENPF in LUAD might be related with its occurrence. At the same time, studies have shown that CENPF mediates mitosis and cell proliferation [22]. The expression of CENPF and DNA content were significantly reduced (Figure 4I), and the cell proliferation was significantly decreased in stably CENPF-deficient LUAD cells (Figure 4C, 4D). These results indicated that low expression of CENPF inhibited proliferation of tumor cells in LUAD. Other studies showed that forkhead box M1 (FOXM1) and CENPF synergistically promoted malignant progression and poor prognosis of prostate cancer [9]. It has been reported that chicken ovalbumin upstream promoter-transcription factor 2 (COUP-TFII) promoted metastasis of prostate cancer through signal transduction of FOXM1 and CENPF [19]. We speculated that the dysregulation of miRNA-COUP-TFII-FOXM1-CENPF axis can be associated with malignant progression, poor prognosis and metastasis in LUAD. Our study revealed that LUAD cells showed a significant reduction in invasion and metastasis after CENPF knockdown (Figure 4E–4H). The expression of CENPF was significantly related to TNM staging of LUAD (Figure 2I–2L; Figure 3B, 3C). In addition, based on the clinical data of LUAD patients obtained from GEPIA and TCGA, LUAD patients with high expression of CENPF was correlated to a poor prognosis (Figure 2N–2P). These results indicated that abnormal expression of CENPF was significantly associated with TNM staging, poor prognosis, and malignant metastasis of LUAD.

In addition, our previous study reported that E2 promoted the progression of lung cancer by binding to ERβ [23]. The biological effects of ERβ in E2 varies based on the targeted organ tumors, including breast, cervical, and prostate cancers [13, 24]. Our previous findings indicated that ERβ2/5 are expressed in lung cancer [12]. Our study results revealed that ERβ2/5 is also highly expressed in LUAD patients (Supplementary Figure 2B, 2D). In addition, ERβ2/5 showed high positive association with the TNM staging of LUAD (Figure 3B, 3C).

CENPF knockdown inhibited the progression of LUAD mediated by the ERβ2/5 pathway. High expression of CENPF and ERβ2/5 is associated with the development of LUAD. Invasion, migration and proliferation of LUAD cells in the CENPF-KD+E2 group showed significant reduction *in vitro* when compared to the CENPF-NC+E2 group (Figure 5C–5H, Supplementary Figure 4A–4F). In the *in vitro* system, the protein expression of ERβ2/5 in CENPF-KD+E2 group was significantly lower than CENPF-NC+E2 group (Figure 5K; Supplementary Figure 5C). In order to eliminate the effects of endogenous estrogen due to gender differences, *in vivo* experiments were only conducted in male mice. Consistent with the *in vitro* experiment results, the expression of ERβ2/5 protein in CENPF-KD+E2 group was significantly lower than that in CENPF-NC+E2 group (Figure 6J; Supplementary Figure 5F). From these results, we confirmed that the knockdown of CENPF inhibited the progression of LUAD mediated by ERβ2/5 pathway both *in vitro* and *in vivo*. This is another important point regarding the mechanism of CENPF, except for FOXM1 [9] and COUP-TFII [19].

Taken together, these findings indicated that both CENPF and ERβ2/5 are highly expressed in LUAD cells and their expression is associated with TNM staging and prognosis in LUAD patients. CENPF knockdown inhibited proliferation, invasion and metastasis of LUAD cells mediated by ERβ2/5 pathway. Thus, the development of inhibitors against the ERβ2/5 subtypes and CENPF can have great therapeutic impact in LUAD. However, there are some shortcomings in this study that should be acknowledged. First, due to the large molecular weight of CENPF, plasmid construction can easily be created off target, thus LUAD cell lines expressing CENPF can be difficult to construct. Second, the number of specimen used for RNA-Seq is limited. These issues are key points that should be targeted for future studies.

## MATERIALS AND METHODS

### Tissue specimens of patient and cell culture

This study was approved by the Ethics Review Committee of Wuhan University. Tissue specimens from 56 LUAD cases and 10 benign pulmonary lesions (BPL) cases who underwent surgery from April 2014 to July 2017 were collected for tissue chip. One pair of LUAD and peri-cancerous tissues were collected for RNA-Seq and three pairs of tissues for profiling protein were obtained from the Department of Thoracic and Cardiovascular Surgery, Zhongnan Hospital of Wuhan University. The tissue chip was customized by Shanghai Core Biotech Co., Ltd. [11]. The isolated tissue samples were immediately stored in liquid nitrogen and sent to Huada Gene (Beijing) for RNA-Seq analysis [4].

Human LUAD cell lines (A549, H1975, H1299 and PC-9) were cultured in RPMI-1640 medium. Normal lung bronchial cells BEAS-2B cultured in DMEM medium were purchased from the Chinese Academy of Sciences cell bank. The medium contained 10% fetal bovine serum (FBS) and double antibody (Gibco, 15140-122).

### Lung cancer patient data set

Training data sets (GSE19804, GSE30219, GSE32863, GSE63459) based on the Affymetrix platform (Affymetrix HG-U133 Plus 2.0 array and HG-U133A array) and corresponding clinical information were retrieved from the Gene Expression Omnibus. Two non-small cell lung cancer (NSCLC) genome-wide expression profiles were extracted from GSE19804 (including 60 paired tumors and normal tissues) and GSE30219 (including 293 tumors and 14 non-tumor tissues). Two LUAD genome-wide expression profiles were extracted from GSE32863 (including 58 paired tumors and normal tissues) and GSE63459 (including 65 tumor tissues).

### Analysis and verification of hub gene

The data sets of GSE19804, GSE30219, GSE32863 and GSE63459 were used to construct co-expression networks and clinical functioning related modules. The genes were screened according to the false discovery rate (FDR) <0.05 and | log2 fold change (FC) |> 1.5. Next, a weighted gene co-expression network analysis (WGCNA) package was used to construct a co-expression network [25, 26]. Finally, the hub gene was selected based on the degree of centrality using the Venn diagram to obtain key genes.

The raw data of RNA-Seq was subjected to quality control, and then mapped with STAR [27] to obtain differential genes. The screening criteria for differential genes were abs(log2FC) > 1 and p value < 0.05.

The Oncomine, Gene Expression Profiling Interactive Analysis (GEPIA) and clinical data from The Cancer Genome Atlas (TCGA) database were used to verify the expression, progression and prognosis of hub gene.

### Immunohistochemistry

The detailed steps for conducting immunohistochemistry was described previously [11]. CENPF (ab5) and ERβ (ab3576) were purchased from Abcam. ERβ1 (MCA1974ST), ERβ2 (MCA2279GT) and ERβ5 (MCA4676T) were purchased from AbDSerotec. The specificity of the above antibodies was confirmed by numerous laboratories including ours [11, 28]. Immunohistochemical method to analyze the optical density was calculated by Image-Pro Plus software.

### Western blotting

Detailed western blotting analysis has been done as described previously [29]. E-cadherin (3195), N-cadherin (13116), MMP2 (40994), CDK2 (2546), CDK4 (12790) and β-actin (4970) were purchased from Cell Signaling Technology. CCND1 (60186-1-Ig) and GADPH (1E6D9) were obtained from Proteintech. The specificity of the above antibodies was verified by numerous laboratories including ours [11, 19, 30].

### Reverse transcription and quantitative real-time PCR (RT-qPCR)

Specific experimental methods were shown in our previously published study [6]. Primers were designed based on CENPF mRNA sequence in GenBank. The primers used were as follows: CENPF, 3- CTCTCCCGTCAACAGCGTTC; CENPF, 5- GTTGTGCATATTCTTGGCTTGC. Data was analyzed using 2^-ΔΔCt^ method. GAPDH, Forward Primer: GGTGA AGGTC GGAGT CAACG; GAPDH, Reverse Primer: CAAAG TTGTC ATGGA TGHACC.

### Cell culture experiment

The sense sequence of CENPF knockdown (KD) and negative control (NC) were integrated into the pWSLV-sh08-GFP vector for transfection of A549 and H1299 cells. The stable CENPF-deficient A549 and H1299 cells were immunofluorescently labeled with anti-CENPF and anti-ERβ [31]. Cell apoptosis assay was conducted using TdT-mediated dUTP-biotin nick end labeling test (TUNEL, Roche Applied Science, Germany) according to the manufacturer's instructions [32]. MTT [33], invasion, migration, scratch and cell cycle experiments were used to evaluate the effects of LV-CENPF sh and LV-NC on the biological function of A549 and H1299 cells [34, 35].

### Xenograft mouse model

Male nude mice were obtained from Beijing HFK Bioscience Co., Ltd., China. Mice were housed in specific-pathogen free environment for one week, and then were subcutaneously injected with 100 μL of 8x10^6^ LV-CENPF sh or LV-NC cells. Tumor size was measured every three days (tumor size = length × width^2^ × 0.5 mm^3^). When the tumor size has reached 80-120 mm^3^, the mice were injected with E2 (0.036 mg/ml, purity 98%, Sigma) or fulvestrant (Ful, 0.800 mg/ml, Sigma) subcutaneously twice a week (6 weeks) [11]. All mice were then sacrificed on day 45, wherein the xenograft tumors were harvested and the tumor weight and size were measured [36]. Tumor tissues were fixed in 4% paraformaldehyde or frozen with liquid nitrogen and stored at -80° C.

### Data analysis

Data are expressed as means ± SD. All analyses were performed at least thrice and the representative data were obtained from three independent experiments. Two-tailed Student's t-test was used to assess significant differences between the groups. The effect of LV-CENPF sh and LV-NC on the biological function of A549 and H1299 cells, the expression of key signaling molecules, immunohistochemistry results in tissue microarray and *in vivo* experiments were analyzed by one-way analysis of variance. Statistical analysis was performed using SPSS 22.0 software (SPSS Inc., Chicago, IL). P<0.05 was considered to be statistically significant.

## Supplementary Material

Supplementary Figures

Supplementary Table 1
