# Supplementary material for: Knockdown of CENPF inhibits the progression of lung adenocarcinoma mediated by ERβ2/5 pathway
Source: Aging (Albany NY). 2021 Jan 10;13(2):2604–25. doi: 10.18632/aging.202303 (PMC7880349; doi:10.18632/aging.202303)
Supplement: Supplementary Figures [file aging-13-202303-s001.pdf]

SUPPLEMENTARY FIGURES

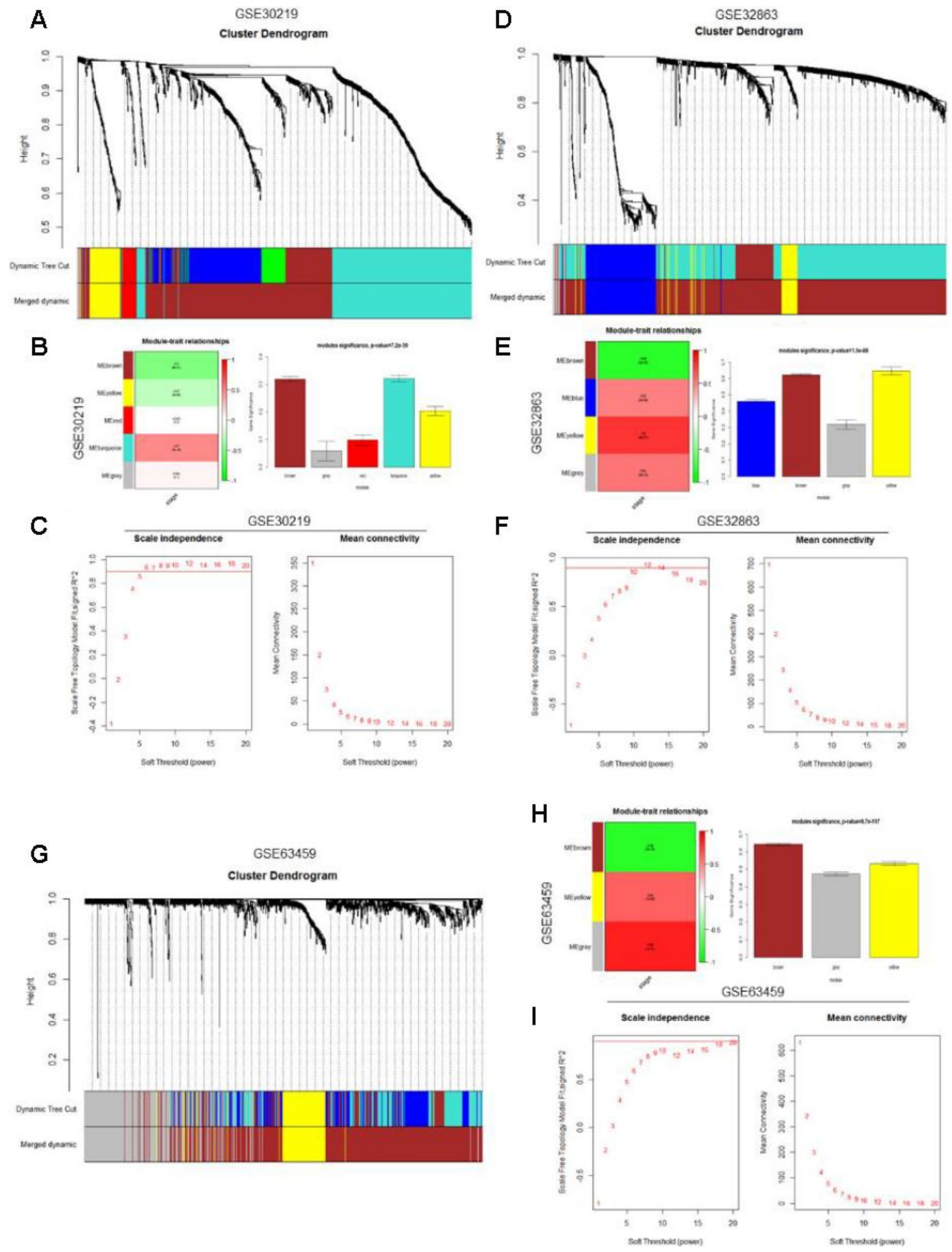

Supplementary Figure 1. WGCNA analysis and determination of the CENPF gene. (**A**, **D**, **G**) Dendrogram of all differentially expressed genes clustered based on a dissimilarity measure (1-TOM) (GSE30219, GSE32863, GSE63459). (**B**, **E**, **H**) Heat maps and distribution of differential genes for different modules related to NSCLC staging (GSE30219) and LUAD staging (GSE32863, GSE63459). (**C**, **F**, **I**) Analysis of the scale-free fit index for various soft-thresholding power (β) and analysis of the mean connectivity for various soft-thresholding power (GSE30219, GSE32863, GSE63459).

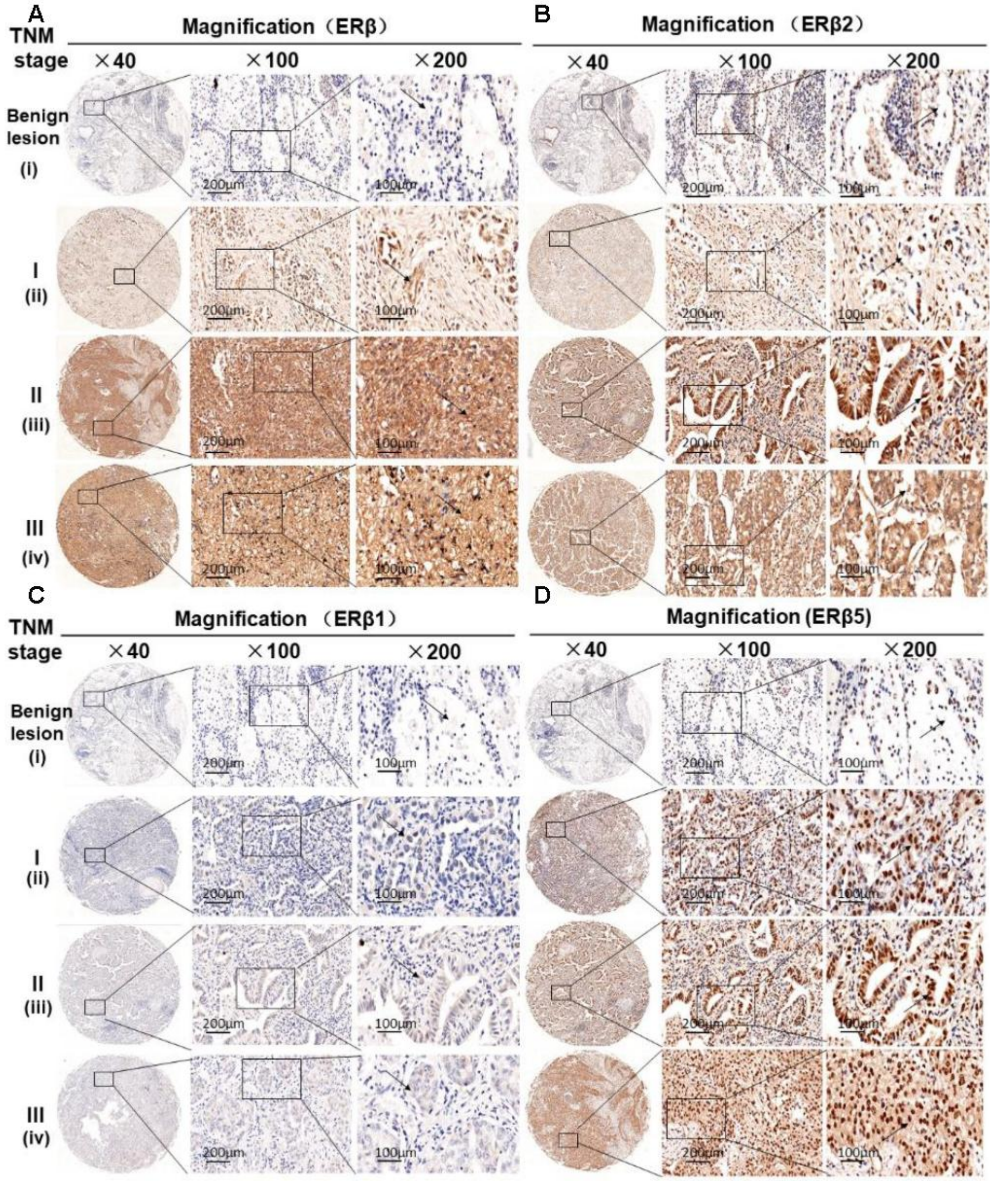

Supplementary Figure 2. (**A**–**D**) Expression of ERβ, ERβ1, ERβ2 and ERβ5 in benign lung lesions (i) and different TNM staging of LUAD (ii=I stage, iii=II stage, iv=III stage). The magnification of each slice is 40×, 100×, 200× in order.

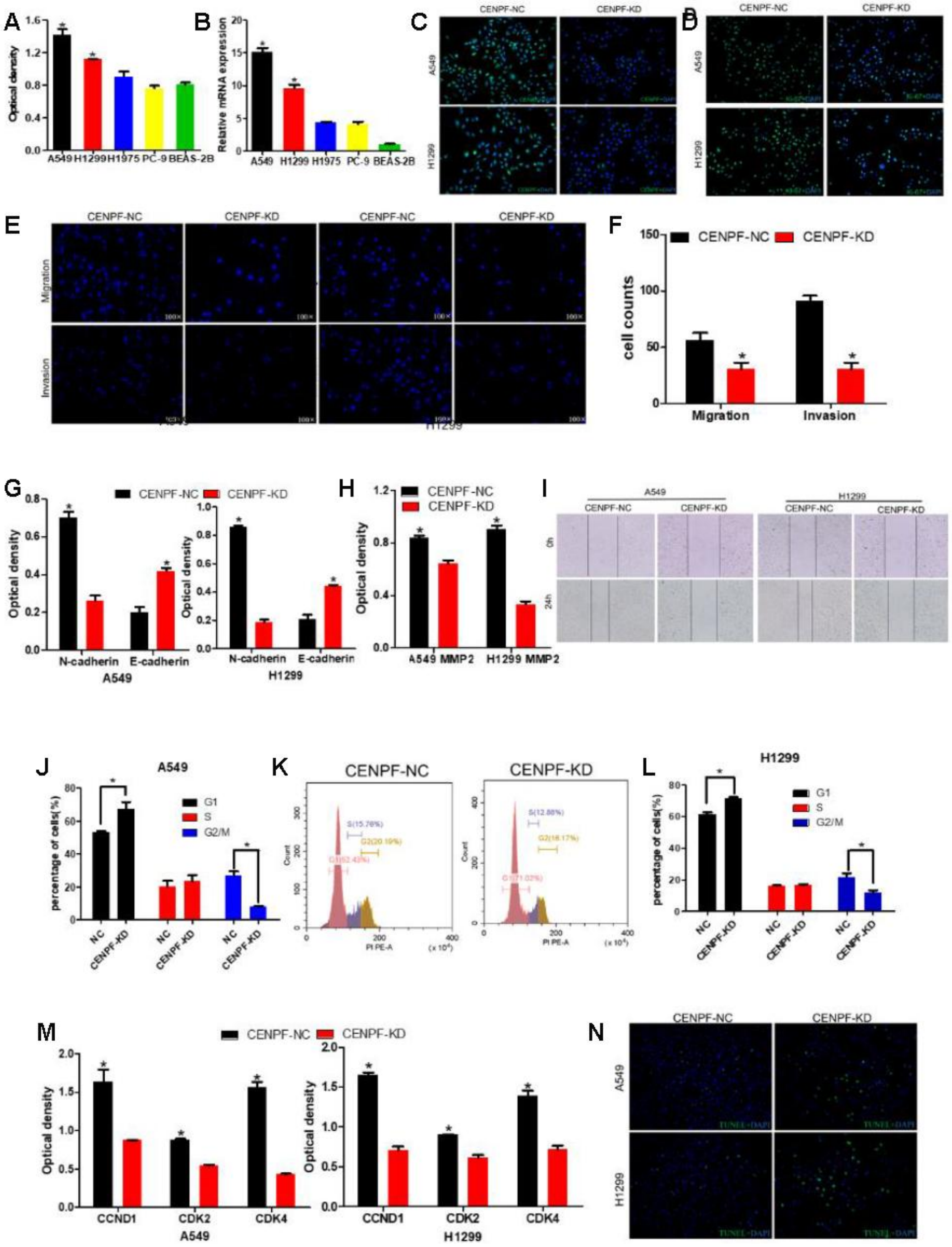

Supplementary Figure 3. Knockdown of CENPF inhibits the biological effects of LUAD cells. (**A**) The protein level of CENPF in A549 and H1299 cell lines were higher than in normal cell lines BEAS-2B and other LUAD cells. GAPDH served as the internal control. *P < 0.05 vs other cells. (**B**) mRNA expression of CENPF in different cell lines. *P < 0.05 vs other cells. (**C**) Representative cellular immunofluorescence images after transfection of CENPF (200×). Green stands for CENPF and blue stands for DAPI. (**D**) Representative Ki67 staining (green) shows cell proliferation of LUAD cells after CENPF-NC or KD treatment (200x). The nuclei were counterstained with DAPI (blue). (**E**, **F**) Migration assays and invasion assays revealed that CENPF-KD decreased cell migration and invasion abilities of A549 and H1299. (**G**, **H**) Corresponding gray value analysis showed that the related protein E-cadherin was significantly increased (P=0.009, Figure 4F), and N-cadherin and MMP2 were significantly decreased when compared with NC group (P=0.004, 0.012) of N-cadherin, E-cadherin and MMP2 in A549 and H1299 cells. (**I**) Representative scratched pictures of A549 and H1299 cells. (**J**–**L**) Percentage of CENPF-KD cells H1299 at different stages of the cell cycle (G1, S and G2/M) and corresponding quantified histograms of A549 and H1299. (**M**) Corresponding gray value analysis showed that the expression of CCND1, CDK2 and CDK4 was significantly lowered in CENPF-KD group (P=0.022, 0.001, 0.002). (**N**) Representative TUNEL staining (green) shows (200x). P values were calculated with two-tailed unpaired Student's t-test, or one-way analysis of variance.

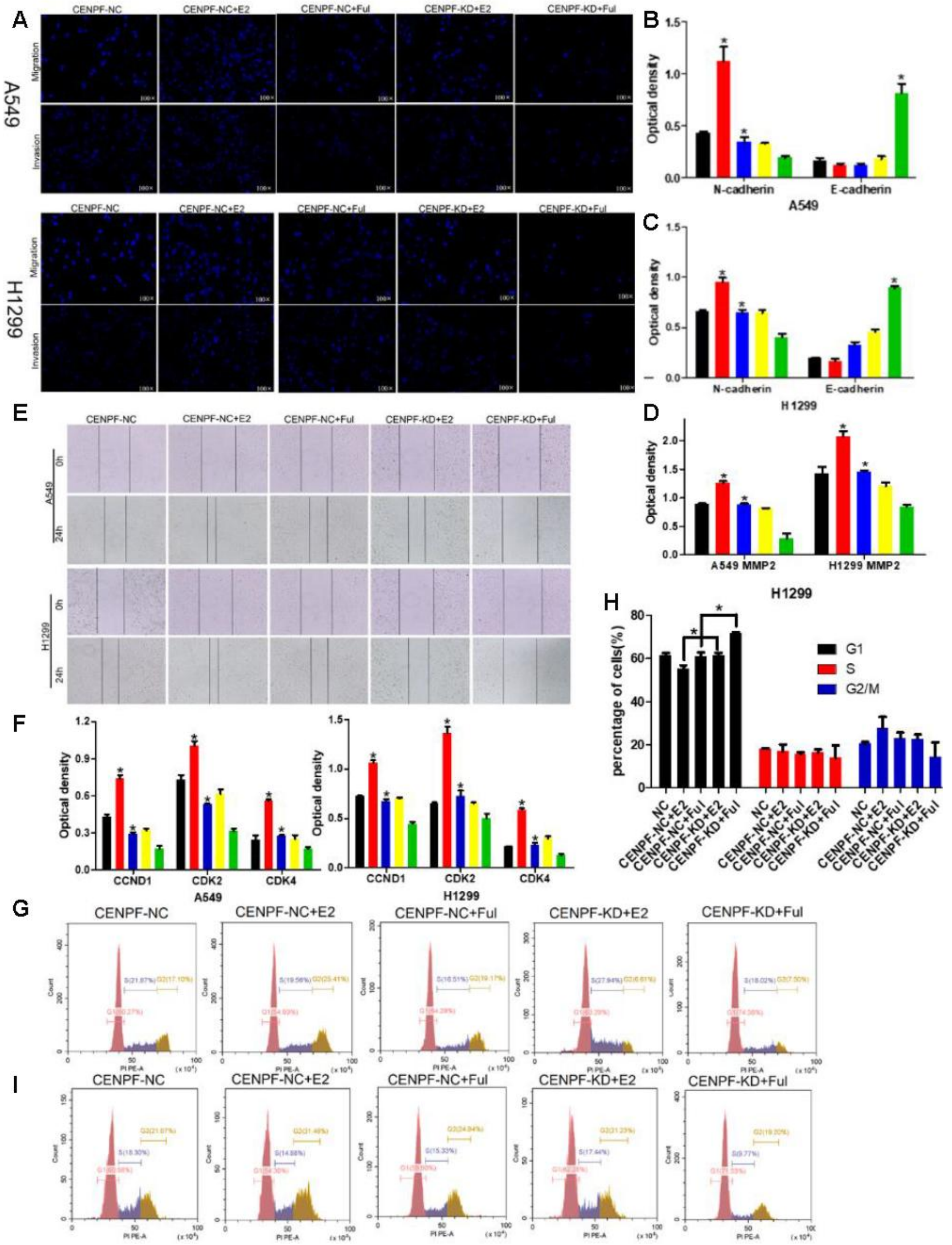

Supplementary Figure 4. Knockdown of CENPF inhibits proliferation, invasion and migration of LUAD cells via the ERβ2/5 pathway. (**A**) Migration and invasion pictures of A549 and H1299 cells. (**B**–**D**) Corresponding quantified histograms of MMP2, N-cadherin and E-cadherin in A549 and H1299 cells. (**E**) Representative scratched images of A549 and H1299 cells. (**F**) Corresponding quantified histograms of CCND1, CDK2 and CDK4 in A549 and H1299. *P < 0.05. (**G**–**I**) Percentage of the A549 cells and H1299 cells at different stages of the cell cycle (G1, S and G2/M) and corresponding quantified histograms. P values were calculated with two-tailed unpaired Student's t-test, or one-way analysis of variance.

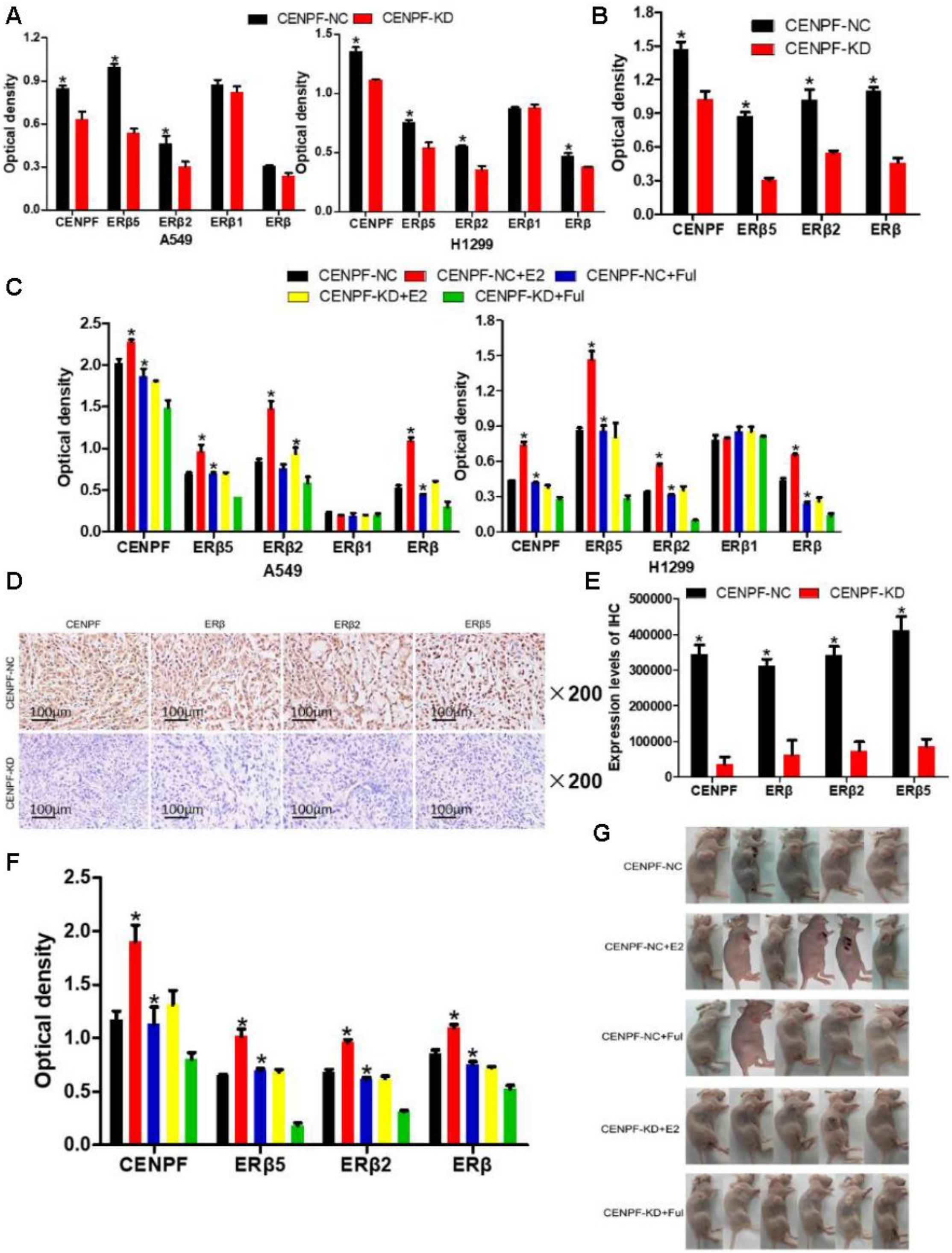

Supplementary Figure 5. Knockdown of CENPF can inhibit the expression of ERβ2/5 *in vitro* and *in vivo*. (**A**, **B**) Corresponding gray value analysis showed that knockdown of CENPF inhibited the expression of ERβ2/5 *in vitro* and *in vivo* experiment. (**C**) Corresponding gray value analysis of CENPF, ERβ, ERβ1, ERβ2 and ERβ5 *in vitro* experiment after treated with E2 and Ful treatment. (**D**, **E**) Immunohistochemical analysis of CENPF, ERβ, ERβ2 and ERβ5 expression in nude mice tumor tissues and corresponding quantitative histograms. *P < 0.05. (**F**) Corresponding gray value analysis of CENPF, ERβ, ERβ1, ERβ2 and ERβ5 *in vivo* experiment after treated with E2 and Ful treatment. (**G**) Pictures of nude mice sacrificed at 45 days. *P < 0.05. P values were calculated with two-tailed unpaired Student's t-test, or one-way analysis of variance.
