# Supplementary material for: Knockdown of CENPF inhibits the progression of lung adenocarcinoma mediated by ERβ2/5 pathway
Source: Aging (Albany NY). 2021 Jan 10;13(2):2604–25. doi: 10.18632/aging.202303 (PMC7880349; doi:10.18632/aging.202303)
Supplement: Supplementary Table 1 [file aging-13-202303-s002.pdf]

SUPPLEMENTARY TABLE

Supplementary Table 1. Genes in key modules.

Hub module	Genes							
Brown	CTHRC1	ARHGAP31	CST1	SUGCT	SORD	PCLAF	TNS1	TNPO1
Module	PAICS	CDCA7	LAMP3	MDK	SFXN1	COMP	TFAP2A	SULF1
(GSE19804)	IGSF9	GJB2	P3H4	UHRF1	CST2	CTTN	MMP12	ATOH8
(n=185)	DNAH14	NMU	PGM2L1	SLC39A8	AQP4	GPX3	CDCA3	SLC46A2
	PEBP4	MS4A15	SOX4	GREM1	KLF9	HSPB8	JPT1	ANP32E
	TENM4	SRPX2	RUNX2	CST4	CPB2	SCG5	E2F8	CENPU
	ACACB	VEPH1	SLC2A1	LRRC15	PCDH7	FIGNL1	MXRA5	MND1
	THY1	ADAM12	ADGRD1	FAM199X	CCDC34	SHCBP1	MKI67	MFAP2
	TRIM59	LRRK2	IQGAP3	FHL2	CILP2	DEPDC1	ABCA3	LAMP5
	FAP	SLC2A5	PLAU	PCP4	PLPP4	SFTA1P	IGF2BP3	DIO2
	KNL1	GPX8	FNDC1	CACNA2D2	DEPDC1B	DEPDC7	PRR11	VCAN
	PLLP	CYP4B1	HOXB7	HLF	WISP1	HOXA10	HSD17B6	FUT9
	GPR87	CILP	CEMIP	SUSD2	SCGB3A2	CDK1	BUB1	CCNB1
	CCNB2	MAD2L1	CDC20	TOP2A	CCNA2	KIF11	BUB1B	DLGAP5
	KIF2C	KIF20A	AURKA	NDC80	CENPF	NUSAP1	BIRC5	NCAPG
	PRC1	TPX2	UBE2C	NUF2	TTK	ZWINT	RRM2	MELK
	CDKN3	PTTG1	CENPK	CHEK1	NEK2	MCM4	ASPM	MCM2
	PBK	KIF4A	CEP55	FOXM1	KIF15	COL1A1	ANLN	COL1A2
	COL10A1	COL11A1	COL3A1	COL5A2	COL5A1	COL8A2	PLOD2	TYMS
	KIF14	ORC6	HMMR	KIF26B	GMNN	FEN1	MMP1	FBXO32
	GINS1	GINS2	GTSE1	ZBTB16	RMI2	UBE2T	ITGA11	BRIP1
	EZH2	CCNE2	PSAT1	PAFAH1B3	MMP11	THBS2	NME1	STIL
	PSPH	FANCI	MMP13	ADAMTS12	ECT2	IGFBP3	SFTPD	PLA2G1B
	SFTPB							
Turquoise	GPD1	BTNL9	SCARA5	PLAC9	GDF10	HSPB6	FHL1	DAPK2
module	TNPO1	FAM189A2	PGR	ANKRD29	DLC1	EMCN	ABCA8	PDE2A
(GSE30219)	CSRNP1	ARHGAP6	LOC100506990	PSMD6-AS2	FHL5	PGM5	NOSTRIN	FILIP1
(n=413)	LTBP4	SPATA13	STARD13	RUFY2	TNS2	PREX2	CFAP70	SYNPO2
	GPX3	IFT57	PCLAF	JCAD	VEPH1	ATAD2	CX3CR1	PALMD
	ADIRF	RBP4	PLPP3	HBB	VAPA	CDCA3	FRY	ATOH8
	TGFBR3	SELENOI	VSIG2	CTHRC1	FAM199X	SRD5A1	HIGD1B	CAB39L
	CDH19	IL33	HMGB3	NR4A1	DIXDC1	NR4A3	MAGI2-AS3	
	ARHGAP44	PLSCR4	ATP5S	COX7A1	UHRF1	SHC3	HLF	2-Mar
	DEPDC1	RNF125	CDO1	HMGB3P1	CKAP2	MMP12	SPARCL1	NDRG2
	FAM13C	NETO2	AUNIP	NTN4	KNSTRN	MKI67	DNAH14	PSAT1
	THY1	E2F8	KNL1	DSP	SPAG5	CRIM1	SIX4	
	PRICKLE2	FIGNL1	RAI2	CCDC34	C1orf112	PIMREG	WFDC1	TMPO
	CENPU	CDCA2	DEPDC1B	SPP1	METTL7A	HELLS	CDCA7	FAM162B
	ECE2	CNRIP1	SAMD4A	HYAL1	ESRP1	NEBL	AFF3	PDK1
	GRAMD2A	DONSON	SGO2	IQGAP3	MYRF	CACNA2D2	RAB11FIP1	CYS1
	CKAP2L	PARPBP	ADAM12	PRR11	PDK4	KL	ABCA3	MLLT11
	COCH	NDC1	DUXAP10	MT1M	PPP1R3C	EPB41L5	SULF1	RERG
	TRIM59	MDK	CGNL1	ARNTL2	ZNF367	NEIL3	RNASE4	8-Sep
	GGH	ADGRD1	KCNE1	SLC12A8	IGSF9	TMEM125	SLC7A5	NEGR1
	CPB2	NR4A2	ID4	IGF2BP3	SLC2A5	CA2	FAXDC2	
	CAMK2N1	PFN2	WASF1	LOXL2	RPL39L	CTSV	PID1	MEST
	CABYR	MTFR2	BCL11A	CDK5R1	CDHR3	C12orf56	MACROD2	CHRNA5
	FAM83D	CRABP2	WISP1	NR3C2	HOXA10	ADAMDEC1	PI15	APOD
	SLC22A3	PRAME	WDR72	TMEM158	FAP	ZIC2	APOBEC3B	FNDC1
	DNALI1	HOXD10	ATP8A1	DLX6	MLPH	CNTNAP2	MAGEA6	CAPN8
	HOXC6	DLX5	HES6	HORMAD1	AQP3	MAGEA12	MAGEA1	TSPAN8
	MS4A8	LGSN	CALB1	CDK1	CCNB1	CCNB2	BUB1	CDC20
	MAD2L1	PLK1	AURKB	CCNA2	CENPE	CDCA8	KIF2C	BUB1B
	NDC80	TOP2A	BIRC5	KIF11	CENPF	KIF18A	CENPL	CENPH
	CENPN	CENPI	CENPK	KIF20A	NUF2	ESPL1	DLGAP5	KIF23
	AURKA	ZWINT	CDCA5	ZWILCH	KNTC1	SPC25	SKA1	PRC1
	UBE2C	CHEK1	NCAPG	TPX2	HIST1H2BD	HIST1H2BH	RACGAP1	TTK
	MCM4	NUSAP1	CDC45	CDC6	MCM2	RFC4	RRM2	POLE2
	PTTG1	CDKN3	ASPM	KIF4A	MELK	SMC2	CDT1	FOXM1
	ORC6	SMC4	NEK2	KIF15	MCM8	CDC25A	SKP2	PBK
	CEP55	MCM10	RAD51	ANLN	PIK3R1	HJURP	OIP5	CENPW
	KIF18B	GMNN	KIF26B	DBF4	TYMS	NCAPG2	EXO1	CCNE1

	ITGA1	NUP155	KIF14	RMI2	FEN1	NCAPH	BRIP1	CDC25C
	AGTR1	HMMR	TIMELESS	COL1A1	CKS1B	UBE2S	MYBL2	COL1A2
	CKS2	COL5A2	PLOD2	LMO7	ADRB2	ZBTB16	ARRB1	GINS2
	CCNE2	RAD51AP1	RNF144B	EZH2	FOS	NR3C1	LMNB1	COL3A1
	CBX2	COL10A1	COL11A1	FBXO5	MMP1	NMU	CTTN	DTL
	EPAS1	GINS1	ECT2	GTSE1	AR	SYT1	SSTR1	GAL
	UBE2T	MND1	P2RY14	CITED2	LPL	SERPINA1	VIPR1	RAMP2
	NME1	RAP1A	CLU	AOX1	MYH11	KPNA2	DUSP1	SORBS1
	FANCI	CBX7	CERS6	ASF1B	NME5	SHCBP1	ALDH2	E2F7
	SGMS2	TRIP13	MAOB	TFAP2A	MMP9	MAOA	SCNN1B	FXYD1
	LRRFIP1	SHANK3	GRIA1	ATP1A2	PAICS	PPAT	PRKCE	
	PAFAH1B3	SUV39H2	FGFR4	GATA2	SCN4B	TPD52	PLA2G1B	
	ADAMTSL3	TSC22D3	MMP11	TK1	GREM1	RAD54B	RNASEH2A	LMNB2
	SLC2A1	IGFBP3	DSCC1	STIL	PGF	THBS2	EGLN3	BMP2
	CDKN2A	HMGA2						
Yellow	CACNA2D2	UHRF1	UBE2T	Pfs2	C9orf140	CDC45L	PGC	
module	MGC24665	C17orf53	ECT2	C1QTNF6	SLPI	CDCA7	SUSD2	WDR51A
(GSE32863)	A2M	HES6	PGCP	PTTG3	HDC	LEPREL1	FLRT3	TBX2
(n=79)	NMU	SCG5	RPL39L	SELENBP1	ZNF533	SCGB3A2	RNASE1	VSIG2
	TRIP13	FOLR1	ANG	KCNK12	C4BPA	IGJ	PCP4	TUBB2B
	AGR3	SCGB3A1	TOP2A	CCNB2	CDC20	AURKB	AURKA	CDCA8
	UBE2C	KIF20A	TPX2	BIRC5	PRC1	NUSAP1	CENPF	PTTG1
	MCM2	NEK2	CDCA5	TYMS	MCM4	MELK	ASPM	NUP155
	TUBG1	FEN1	TIMELESS	CCNF	KIFC1	KIF1A	STIL	TK1
	H2AFX	KIAA0101	ALDH2	E2F2	SFTPD	CCNE1	MAOA	SFTPB
Yellow	ADRB2	MARCKSL1	CES1	IDH2	CSTF2	PSAT1	C6orf125	NOSTRIN
module	KIAA0859	ST6GALNAC6		STOM	PLA2G4F	WFS1	MAL	XPO5
(GSE63459)	SAP18	TNFSF12	MTA3	MGC13170	NARF	SLC2A1	C17orf53	CALM1
(n=160)	GSN	CDC45L	MOCS1	ALG8	TSPAN4	C1orf112	TLE4	EDNRA
	C6orf129	IFT57	WDR51A	CIRBP	AHCY	TMEM132A	C20orf20	
	MGC24665	MS4A2	GDDR	TPSAB1	HMGA1	BOLA2	ECRG4	FLJ40629
	HCAP-G	CABYR	PCCB	NUDT1	GPR116	PLOD1	C9orf140	SUSD2
	NCALD	SLPI	C20orf24	ABCA3	VSIG2	PTTG3	KLF2	DLG7
	TROAP	PGCP	TMEM106C	CPA3	SFTPD	MSN	DLC1	
	DKFZp762E1312	SELENBP1	CDC2	NR3C2	HES6	C16orf60	RAFTLIN	C18orf56
	C1orf116	RPS6KA2	HLA-DRB6	CTSH	RNASE1	IGF2BP3	FOXA2	BUB1
	CCNB2	CCNA2	CDC20	AURKB	TOP2A	CDCA8	AURKA	KIF11
	CENPF	KIF2C	KIF20A	TPX2	BIRC5	PRC1	CHEK1	NUSAP1
	UBE2C	CENPM	MCM3	MCM4	MCM2	RFC4	KNTC1	CDCA5
	SPC24	PTTG1	RCC2	TTK	CDKN3	NEK2	PLK4	MCM7
	MELK	CEP55	FOXM1	ASPM	TYMS	MCM6	E2F3	FEN1
	HMMR	TIMELESS	MCM10	ANLN	CDK4	E2F2	EXO1	TUBG1
	CKS1B	CCNE1	CCNF	EZH2	RAB3IP	KIAA0101	RAD51AP1	POLQ
	OIP5	SHMT2	UBE2T	TK1	STIL	CBX2	ALDH2	PAICS
	CCT3	GAPDH	APOA1BP	MRPL12	CD59	RPL34	MRPS17	RPL39L
	TRIP13	MAOA	FOLR1					
